# Rapid molecular detection of macrolide resistance

**DOI:** 10.1186/s12879-019-3762-4

**Published:** 2019-02-12

**Authors:** Megan M. Nelson, Christopher L. Waldron, John R. Bracht

**Affiliations:** Department of Biology, American University, Washington, DC 20016 USA

**Keywords:** Recombinase polymerase assay, Antimicrobial resistance, Point-of-care diagnostics

## Abstract

**Background:**

Emerging antimicrobial resistance is a significant threat to human health. However, methods for rapidly diagnosing antimicrobial resistance generally require multi-day culture-based assays. Macrolide efflux gene A, *mef(A)*, provides resistance against erythromycin and azithromycin and is known to be laterally transferred among a wide range of bacterial species.

**Methods:**

We use Recombinase Polymerase Assay (RPA) to detect the antimicrobial resistance gene *mef(A)* from raw lysates without nucleic acid purification. To validate these results we performed broth dilution assays to assess antimicrobial resistance to erythromycin and ampicillin (a negative control).

**Results:**

We validate the detection of *mef(A)* in raw lysates of *Streptococcus pyogenes, S. pneumoniae, S. salivarius,* and *Enterococcus faecium* bacterial lysates within 7–10 min of assay time. We show that detection of *mef(A)* accurately predicts real antimicrobial resistance assessed by traditional culture methods, and that the assay is robust to high levels of spiked-in non-specific nucleic acid contaminant. The assay was unaffected by single-nucleotide polymorphisms within divergent *mef(A)* gene sequences, strengthening its utility as a robust diagnostic tool.

**Conclusions:**

This finding opens the door to implementation of rapid genomic diagnostics in a clinical setting, while providing researchers a rapid, cost-effective tool to track antibiotic resistance in both pathogens and commensal strains.

## Background

Combating antimicrobial resistance (AMR) is a national and international priority. The U.S. National Institutes of Health [1], Center for Disease Control [2], World Health Organization [3], and United Nations [4] have prioritized the issue. On Sept. 18, 2014 former President Barack Obama issued AMR-focused Executive Order 13676 [5], which was followed by a National Action Plan for Combating Antibiotic Resistant Bacteria [6].

However, surveillance of antimicrobial resistance is a significant challenge [3, 6, 7], causing difficulties in obtaining a realistic threat measurement [3, 6], and impairing the ability to form future projections [8]. Current methods of assessing antimicrobial resistance are extremely slow, requiring days to weeks of culture time, and are also costly in terms of laboratory materials and technician effort [9]. Correspondingly, they are deployed unevenly, biasing our estimates of AMR worldwide and inhibiting our ability to accurately assess this threat to human health [8]. Responding to calls for new diagnostic methods to address this unmet need [7], here we report a simple, rapid, culture-free genomic method for detecting antimicrobial resistance within 10 min of assay time. We also validate a simple raw-lysate preparation method that does not require nucleic acid purification. Together these innovations address a critical need in surveillance of antimicrobial resistance.

Recombinase Polymerase Amplification (RPA), an isothermal alternative to Polymerase Chain Reaction (PCR), uses recombinase-primer complexes to identify and denature the genomic segment of interest, along with single-stranded DNA-binding proteins to stabilize the open DNA [10]. Detection is similar to Taq-Man hydrolysis probes [11] except that the probe contains an internal abasic site analog, tetrahydrofuran, that is cleaved by Endonuclease IV (*nfo*) [12] during the course of amplification [10]. The polymerase used is strand-displacing *Bsu* [10], which is more resistant to chemical inhibition than Taq, giving RPA more robustness than PCR [13]. Because DNA denaturation is performed by proteins rather than heat, RPA occurs isothermally, usually 37 °C - 42 °C, and multiple reports document improved speed for RPA relative to PCR, often with detection within 5–7 min [13–15]. In addition, RPA demonstrates extreme sensitivity, often detecting tens of copies of a nucleic acid target [10, 14–17]. While RPA has not been widely implemented in clinical settings, it has been proven capable of detecting bacterial, viral, and protozoan human pathogens. Eukaryotic pathogens detected with RPA include the blood-fluke *Schistosoma japonicum* [15] and the diarrheal protozoan pathogens *Giardia*, *Cryptosporidium*, and *Entamoeba* [17, 18]. Viral pathogens detected by RPA include HIV [19, 20], Chikungunya virus (CHIKV) [14], Rift Valley Fever virus [21, 22], Middle East respiratory syndrome coronavirus [23], foot-and-mouth disease virus (FMDV) [24], Bovine Coronavirus [25], and Crimean-Congo Haemorrhagic fever Virus (CCHFV) [26]. Bacterial pathogens detected by RPA include *Mycoplasma tuberculosis* [27, 28], *Neisseria gonorrhoeae*, *Salmonella enterica,* and methicillin-resistant *Staphylococcus aureus* (MRSA) [29], *Chlamydia trachomatis* [30], *Francisella tularensis* [31], Group B *Streptococci* [32], *Orientia tsutsugamushi* (scrub typhus), and *Rickettsia typhi* (murine typhus) [16].

In diagnostic applications RPA has been shown to be highly specific and thus resistant to false positives (Type I errors). In several cases 100% specificity was shown [14–16, 20]. Because of the health risks of erroneous detection and treatment, high specificity is an important characteristic of diagnostic assays. Type II errors (false negatives) are always possible if the pathogenic target is present at a low level in a sample, but the exquisite sensitivity of RPA (see above) minimizes this risk.

In this study, we developed and tested a novel RPA assay for the detection of the Macrolide Efflux A, or *mef(A)* gene, an efflux pump rendering host bacteria resistant to 14- and 15-membered macrolide antibiotics (including erythromycin A and azithromycin) [33, 34]. This gene can be found within *Streptococcus pyogenes,* the largest member of the Lancefield group A streptococci, where it is encoded on a transposon that is integrated into a prophage [35, 36]. While initially identified in *S. pyogenes* and *S. pneumoniae* [33] it has since been identified in an extremely wide range of gram-positive and negative bacteria worldwide [37] consistent with horizontal transfer of antimicrobial resistance genes.

Using purified DNA, a panel of bacteria cultures, and broth dilution antimicrobial resistance testing, we demonstrate extreme sensitivity and specificity of the RPA assay, and we confirm that positive results correctly predict antimicrobial resistance. Our RPA assay uncovered an unexpected occurrence of the *mef(A)* gene within commensal *Streptococcus salivarius* strain, and subsequent laboratory testing confirmed that this strain has genuine antimicrobial resistance. While *S. salivarius* has been known to frequently harbor antimicrobial resistance genes [38], this is the first case, to our knowledge, of antimicrobial resistance first discovered by RPA and confirmed by more traditional methods.

## Methods

### Bacterial strains

*Streptococcus pyogenes* strains MGAS 10394 (ATCC BAA-946) and MGAS 6180 (ATCC BAA-1064), were obtained directly from ATCC (Manassas, VA). *Streptococcus agalactiae* (NR-44140), *S. pneumoniae* GA17457 (NR-19118), *S. pneumoniae* GA16242 (NR-19111), *S. pneumoniae* NP112 (NR-19213) and *E. faecium* Strain 513 (HM-959) were obtained from beiresources.org (Manassas, VA). *Streptococcus salivarius* was isolated by the Kaplan lab of American University (Washington, DC) with IRB approval and patient consent for research.

Presence or absence of *mef(A)* and *ermB* genes were assessed by local blastn against published genomes downloaded from the following GenBank accessions: *S. pyogenes* MGAS10394, accession CP000003.1; *S. pyogenes* MGAS6180, accession CP000056.1; *S. pneumoniae* strain GA17457, accession AILS00000000.1; *S. pneumoniae* GA16242, accession AGPE00000000.1; *S. pneumoniae* strain NP112 accession AGQF00000000.1; *S. agalactiae* SGBS025, accession AUWE00000000.1; and *Enterococcus faecium* Strain 513 accession AMBG00000000.1.

### Antibiotic testing by broth dilution

*S. pyogenes, S. agalactiae,* and *S. salivarius* were tested for their antimicrobial susceptibility by broth microdilution. Ampicillin (Cat # 97061–442) was obtained from VWR (Amresco) and Erythromycin (Cat # TCE0751-5G) was obtained from VWR (TCI). Bacteria were maintained on blood agar plates at 37 °C, and single colonies selected for inoculation into liquid overnight cultures in sterile Brain-Heart Infusion (BHI, VWR Cat # 90003–038). For each culture, 14 ml of BHI media was inoculated in a sealed 15 ml falcon tube for overnight incubation at 37 °C (no shaking). Gentle inversion was used to mix the cultures prior to setting up the assay.

For the experiment, 5 μl of overnight culture was mixed with 5 ml of BMI media (1000x dilution) in a sterile tray and gently mixed. This dilute culture was added at 180 μl per well of a 96-well plate pre-loaded with 20 μl of antibiotic solutions ranging, for erythromycin, from 0.5 to 32 μg/ml (10x) to produce the desired final concentrations of 0.05–3.2 μg/ml. For ampicillin, the stocks were 1.25 μg/ml-80 μg/ml resulting in final concentrations of 0.125 μg/ml-8 μg/ml. The 96-well plate was then transferred to a FilterMax F5 microplate reader for a 20 h incubation at a temperature of 37 °C, with readings taken every 30 min. A 10-s orbital shaking was performed prior to each reading.

### Specificity testing & adipose-derived stem cell culture

For specificity testing, human DNA was derived from primary adipose-derived cell line ASC080414A (commercially obtained from Zen-Bio, Raleigh, NC) cultured in a humidified 5% CO2 incubator at 37 °C. The growth media consist of Dulbecco’s Modified Eagle Medium (DMEM, ThermoFisher # 11965118) supplemented with 10% fetal bovine serum (ThermoFisher # 10082147), 1X Penicillin / Streptomycin (ThermoFisher # 15140122), and 1X Glutamax (ThermoFisher #35050061), changed every 3 days. Total DNA was purified using the Nucleospin Tissue kit (Macherey-Nagel, Düren, Germany) and quantified on a Qubit Fluorometer (ThermoFisher), which was also used to measure bacterial DNA liberated in crude lysates.

### RPA assays

Primers and probe for the *mef(A)* RPA assay (Table 1) were designed following the instructions provided by TwistDx (Cambridge, UK). All primers and probes were synthesized by Integrated DNA Technologies (Coralville, Iowa). For all RPA assays the TwistDx *nfo* kit (TANFO02KIT, TwistDx, Cambridge, UK) was used in agreement with manufacturer’s instructions. For each reaction, a hydration mix was prepared including 4.2 μl of RPA primer pair (2.1 μl of each 10 μM primer), 0.6 μl of Probe (10 μM), 29.5 μl of rehydration buffer, and 13.2 μl of sample containing DNA or lysate to be tested (47.5 μl total). Then the hydration mix was added to a reaction tube containing TwistAmp lyophilized enzyme pellet. The resulting mixture was mixed via pipetting 3–4 times carefully to avoid introduction of bubbles, and transferred to a qPCR 96-well plate (Agilent Cat # 410088). Final concentration of primers was 420 nM and the probe was 120 nM. To activate the reaction, 2.5 μl of magnesium acetate stock solution (280 mM) was added to the caps of the 96-well plate, rapidly mixed via inversion, immediately placed in a qPCR machine (Agilent Stratagene Mx3005P). The reaction was maintained at constant temperature of 37 °C for 30 min, with FAM signal recorded every 30 s (60 total readings).

**Table 1:** Primers and probes used in this study

Name	Sequence
mefA_RPA_F1	5’-GCGGTTACGCCACTTTTAGTACCAGAAGAACAGCT-3’
mefA_RPA_R1	5′-[Biotin]-TTTAGTTCCCAAACGGAGTATAAGAGTGCTGCAAC-3’
mefA_RPA_probe	5′−/56-FAM/CAGGCTATAGTCAGTCTTTGCAGTCTATAAGC/idSp/ATATTGTTAGTCCGGC/3IABkFQ/− 3’
27F	5’-AGAGTTTGATCCTGGCTCAG-3’
388R	5’-TGCTGCCTCCCGTAGGAGT-3’

### qPCR assay

Primers F1 and R1 (Table 1) were combined at a final concentration of 176 nM with control DNA (MGAS10394) dilutions at indicated concentrations, in 1X PowerSYBR (ThermoFisher Cat # 4367659) and run on an Agilent Stratagene Mx3005P. We used a 2-step program with 40 cycles of 30 s at 95 °C and 1 min at 60 °C. The total program time was 2 h 16 min.

### PCR: 16S rDNA and *mef(A)*

Bacterial identification was carried out using primers 27F and 388R with 2 μl raw lysates prepared by boiling and diluting the overnight cultures. Amplification was performed in a SimpliAmp thermocycler (Applied Biosystems) with a program of 32 cycles with 95 °C for 30 s, 52 °C for 30 s, and 72 °C for 25 s.

Detection of *mef(A)* was performed by PCR using F1 and R1 primers and 2 μl raw lysates as above. The program used was 30 cycles of 95 °C for 30 s, 60 °C for 30 s, and 72 °C for 10 s.

## Results

**Fig. 1:**
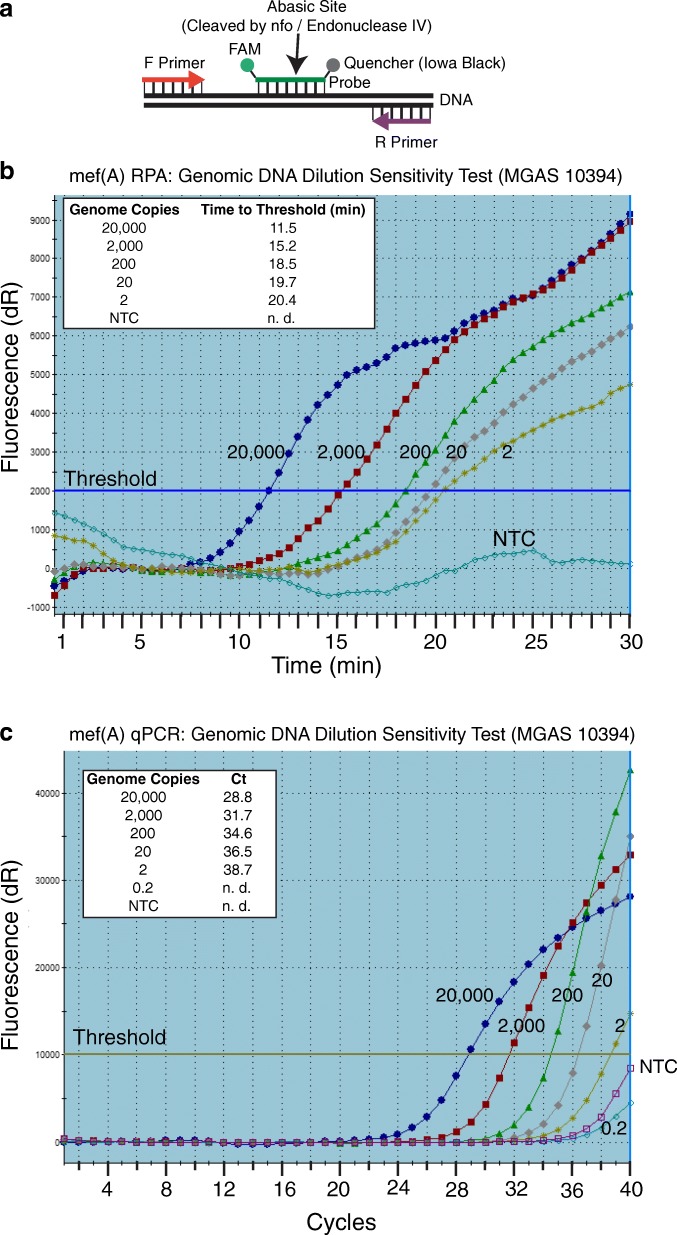
Design and sensitivity testing of Recombinase Polymerase Assay (RPA) against *mef(A)* gene. **a** Schematic of probe and primer design. Taq-Man-style hydrolysis probe is cleaved by *nfo* endonuclease during amplification, releasing the quencher and activating FAM signal. Quencher serves as 3′ blocking moiety. **b** RPA sensitivity testing using serial dilutions of DNA from *mef(A)*-positive *Streptococcus pyogenes* strain MGAS10394. **c** Comparison with qPCR using the primers from RPA (**b**), but using Sybr Green as readout instead of FAM (the probe was not used)

We designed a Taq-Man style hydrolysis probe incorporating fluorophore (FAM) and quencher (Iowa Black) which doubles as a 3′ end blocker. Successful amplification leads to probe cleavage by Endonuclease IV (*nfo*) at the abasic site, separating FAM from the quencher and yielding detectable signal. Earlier work used a quencher and FAM internally, proximal to the abasic site [10]; our design simplifies this by using the quencher as a 3′ end blocker (Fig. 1a).

**Fig. 2:**
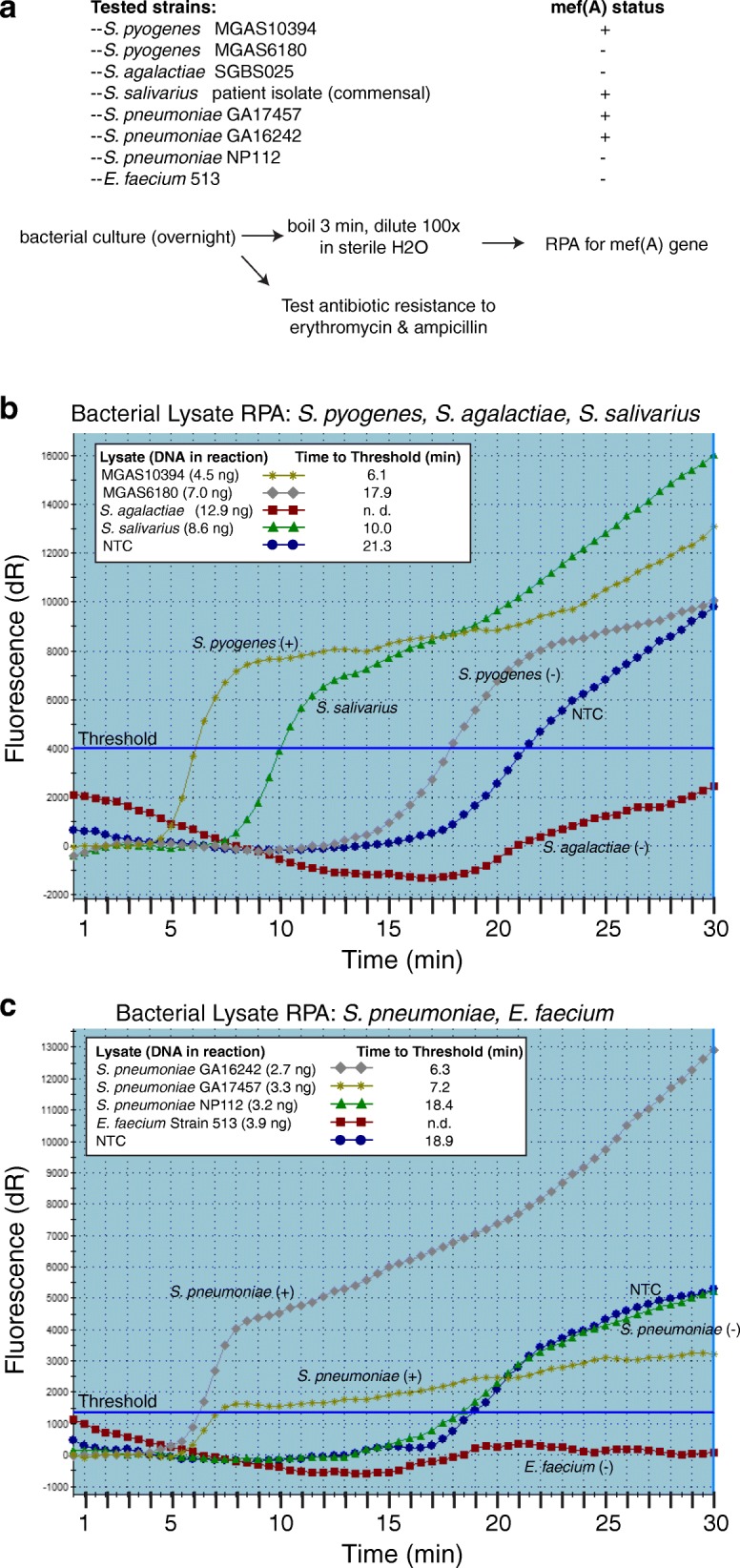
Bacterial panel for RPA assay and validation of raw lysate method. **a** Schematic of culture and bacterial lysate method. **b** *Mef(A)* RPA results for *S. pyogenes, S. agalactiae,* and *S. salivarius*. **c** *Mef(A)* RPA results for *S. pneumoniae* and *E. faecium*. For panels **b** and **c**, DNA concentration in raw lysates was measured and total amount of DNA loaded into each reaction is indicated, and lines are labeled with species name and whether they are known *mef(A)* positive (+) or negative (−)

To assess assay sensitivity we ran a serial dilution of DNA derived from *mef(A)*-positive *Streptococcus pyogenes* serotype M6 strain MGAS10394 [39] and found that confident detection was around 2000 genome copies (Fig. 1b). Two-thousand genome copies corresponds to 4.3 picograms (pg) of DNA, at a concentration of 252 femtomolar (fM). While the FAM signal crosses the threshold for 200, 20, and 2 genome copies, these signals are probably nonspecific as demonstrated by negative controls showing similar late-rising (around 20 min or later) signal (Figs. 2b, c, and 5). We conclude that the confident sensitivity limit of our assay is approximately 2000 genome copies, and that detection must be recorded before 16 min to be considered real. The non-specific 18–20-min signal was always easily distinguishable from real detection in our assays, which always came up quickly, around 7–10 min (compare Figs. 2b, c, and 5). We suggest the late-rising signal is analogous to qPCR’s tendency to ubiquitously amplify even no-template controls by 40 cycles. We performed SYBR green based qPCR on the same DNA dilution series using the same primers, and observed even greater sensitivity—relatively confidently down to 20 genome copies—but it was significantly slower –the run took over 2 h (Fig. 1c). As discussed later, the 2000 genome copy threshold may help distinguish diagnostically meaningful *mef(A)* gene loads, rather than mere colonizers [40].

**Fig. 3:**
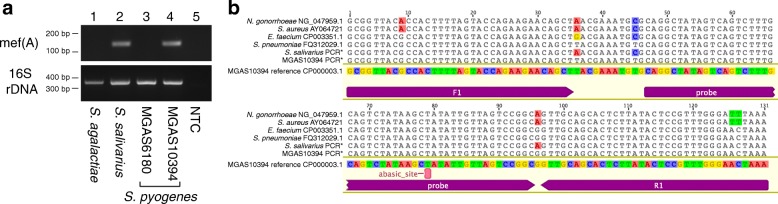
Confirmation of *mef(A)* gene in *Streptococcus salivarius* by PCR and sequencing. **a** PCR against *mef(A)* was performed with the RPA primers (Table 1). 16S rDNA was amplified as a loading control with universal bacterial primers 27F and 388R (Table 1). **b** Alignment showing that *S. pyogenes* and *S. salivarius mef(A)* genes are different. MGAS10394 reference CP000003.1 is set as reference and differences are highlighted in figure. PCR-derived sequences are marked with an asterisk

**Fig. 4:**
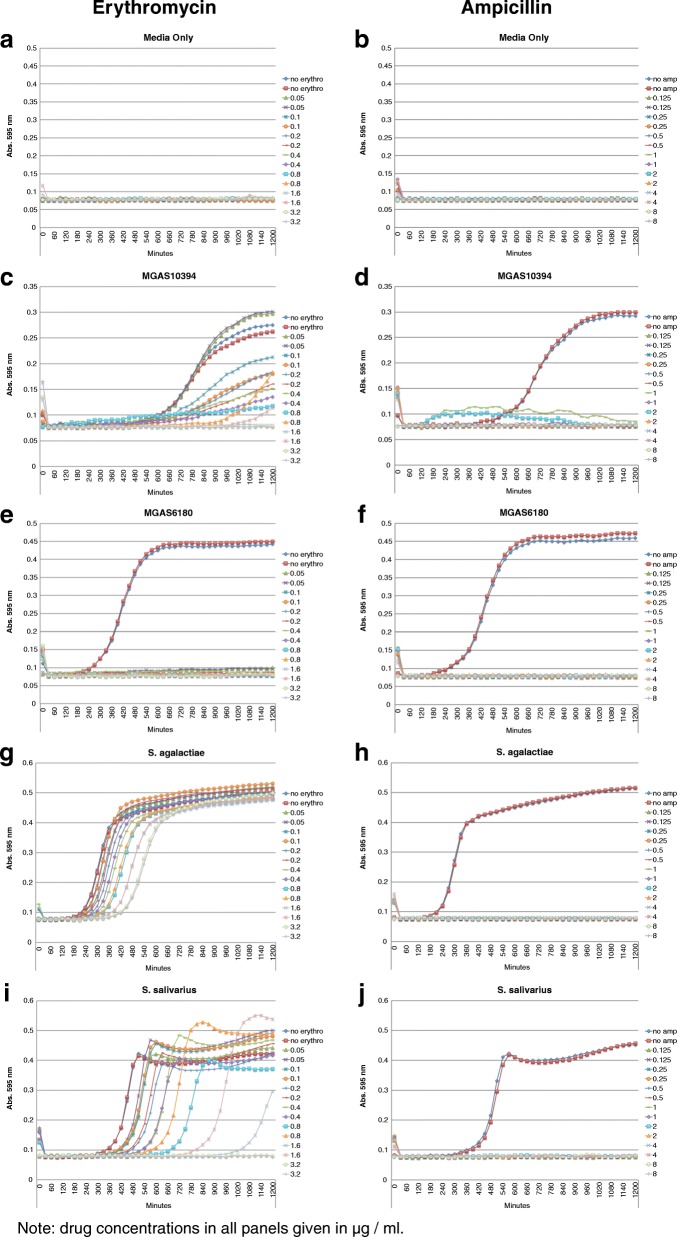
Antibiotic testing to confirm erythromycin resistance in *S. salivarius,* MGAS10394, and *S. agalactiae*. Ampicillin (always the second panel) serves as negative control (all strains susceptible). **a + b,** Media only. **c + d,** MGAS10394 (*mef(A)* positive). **e + f,** MGAS6180 (*mef(A)* negative). **g + h,** *S. agalactiae* (*ermB* positive and *mef(A)* negative). **i + j,** *S. salivarius* (*mef(A)* positive)

We next performed specificity testing with raw bacterial lysates from eight bacterial strains. *Mef(A)* is present within the genomes of Group A Strep strain *S. pyogenes* MGAS10394 [39] and *S. pneumoniae* strains GA17457 and GA16242. Known *mef(A)* negative strains include *S. pyogenes* MGAS6180 [41] responsible for necrotizing fasciitis and puerperal sepsis, *Enterococcus faecium* Strain 513, *S. pneumoniae* strain NP112, and *S. agalactiae* SGBS025. *Streptococcus agalactiae* is resistant to macrolides by a different mechanism than *mef(A)*: it hosts a target-site ribosomal methylase, *ermB.* Methylation of the target site in the 23S rRNA by *ermB* inhibits the interaction of antibiotic with the ribosome [42]*.* We therefore predicted—and confirmed—that this species would show an absence of *mef(A)* by RPA but nonetheless display robust resistance to erythromycin (Fig. 4g). Finally, we tested a patient isolate of *S. salivarius* with an unknown *mef(A)* status. The identities of *S. salivarius, S. agalactiae,* and *S. pyogenes* strains were confirmed by sequencing the 16 s rDNA locus.

We developed a simple raw lysis method. Individual bacterial colonies were inoculated into BHI media for overnight incubation at 37 °C, followed by lysis by boiling at 95 °C for 3 minutes and 100-fold dilution into sterile H_2_O. RPA was performed directly on this raw lysate (Fig. 2a). We tested eight bacterial strains in total: *S. pyogenes (2 strains), S. agalactiae, S. salivarius, S. pneumoniae (3 strains),* and *E. faecium.* RPA confirmed the presence of *mef(A)* within all known positive strains and none of the known negatives (Fig. 2b, c). RPA indicated the presence of *mef(A)* within *S. salivarius,* an unexpected result (Fig. 2b). While we had not expected this commensal species to contain *mef(A)*, we nevertheless performed PCR which confirmed the gene’s presence in MGAS10394 and *S. salivarius* (Fig. 3a). By Sanger sequencing this product we observed that the *S. salivarius* gene has three single-nucleotide polymorphisms (Fig. 3b), suggesting that it has acquired a more divergent copy of the gene and confirming that the detections constitute independent *mef(A)* genes, not cross contamination.

To test whether the *mef(A)* gene is functional, we performed broth dilution of both strains of *S. pyogenes, S. salivarius, and S. agalactiae* with erythromycin and ampicillin (a negative control) (Fig. 4). This confirmed that *S. pyogenes* MGAS10394, *S. agalactiae,* and *S. salivarius* are all resistant to erythromycin (MIC greater than or equal to 3.2 μg/ml, Table 2) and MGAS6180 is susceptible (Fig. 4). As reported by others, *ermB* gives stronger erythromycin resistance than *mef(A)* [43, 44], with *S. agalactiae* giving a MIC > 3.2 μg/ml (Table 2). All tested strains were susceptible to ampicillin as expected (Fig. 4, Table 2).

**Table 2:** Summary of RPA, PCR, and resistance data for bacterial strains. n.d., test not performed

Species	Strain	*mef(A)* RPA from lysate (min)	PCR	MIC (μg/ml) (erythromycin)	MIC (μg/ml) (ampicillin)	Notes
*S. pyogenes*	MGAS10394	6.1	+	3.2	< 0.125	known mef(A) +
*S. pyogenes*	MGAS6180	17.9	–	< 0.05	< 0.125	known mef(A) -
*S. agalactiae*	SGBS025	not detected	–	> 3.2	< 0.125	known mef(A) - and ermB +
*S. salivarius*	Patient isolate	10.0	+	3.2	< 0.125	discovered mef(A) +
*S. pneumoniae*	NP112	18.4	n.d.	n.d.	n.d.	known mef(A) -
*S. pneumoniae*	GA17457	7.2	n.d.	n.d.	n.d.	known mef(A) +
*S. pneumoniae*	GA16242	6.3	n.d.	n.d.	n.d.	known mef(A) +
*E. faecium*	Strain 513	not detected	n.d.	n.d.	n.d.	known mef(A) -

**Fig. 5:**
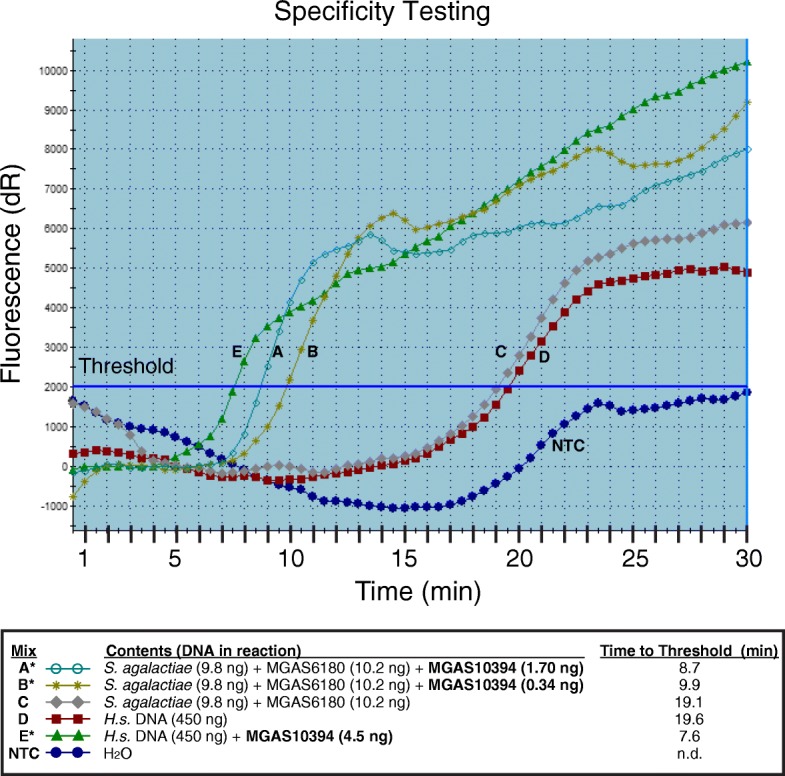
Specificity testing using combined raw bacterial lysates and spiked-in purified human genomic DNA. *H.s.* DNA derived from human adipose-derived stem cells. Mixes positive for *mef(A)* are indicated with an asterisk and the *mef(A)-*containing lysates indicated in bold along with the total DNA in the mixture

To evaluate assay specificity we constructed mixtures of nucleic acids as follows: A, B, and C contain 20 ng of DNA from non-*mef(A)* lysates (*S. agalactiae* plus MGAS6180) either by themselves (C) or spiked with 1.7 ng (A) or 0.34 ng (B) of MGAS10394 (*mef(A)-*positive). Mixes A and B represent 7.8 and 1.7% *mef(A)* positive, respectively. Mixes D and E tested the effect of human DNA, which might be expected to contaminate clinical samples. We therefore tested either 450 ng human DNA alone (D) or with 4.5 ng (1%) of *mef(A)*-positive MGAS10394 lysate (E). None of the non-specific DNA had any apparent effect on the reactions, with only E, A, and B giving specific signal and in proportion to the total *mef(A)* gene present in the samples (4.5 ng, 1.7 ng, and 0.34 ng, respectively) (Fig. 5). The *mef(A)-*negative C and D samples yielded no specific signal, giving non-specific time-to-threshold of 19.1 and 19.6 min, respectively (Fig. 5). Not only do these results show that the RPA assay was 100% specific and quantitative in the presence of non-specific DNA, but also functions with a wide range of total DNA in the mixture (from a few picograms, Fig. 1b, to 450 ng, Fig. 5), and is robust to the conditions of raw lysate including denatured proteins, lipids, and cell wall debris.

## Conclusions

Genomic diagnostics offer the flexibility to in principle detect genetic material in any pathogen—bypassing the challenges associated with antibody-based assays which are much more cumbersome to produce while also being less sensitive than nucleic-acid based methods. For example, two meta-analyses of the rapid antigen-based test for group-A Streptococcal pharyngitis found an 86% sensitivity [45, 46], so 14% of true positives are missed by this method. Here we demonstrate a simple RPA-based genomic procedure offering flexibility and rapid detection within a similar timeframe as the rapid tests (10–15 min) that is suitable to a point-of-care application. We show that we can detect down to the femtomolar (fM) / picogram (pg) range (Fig. 1b). We found that spiking in up to 100x more non-specific DNA than *mef(A) +* DNA did not inhibit the assay, which remained extremely quantitative and specific to true target levels (Fig. 5).

Detection of antimicrobial resistance genes has been more frequently performed with loop-mediated isothermal amplification (LAMP) rather than RPA. Examples include detection of the beta-lactamase responsible for carbapenem resistance in *Acinetobacter baumannii* [47, 48], the class 1 integron-integrase gene *intI1* from environmental samples [49], *msrA* from *Staphylococcus aureus* [50] and *mcr-1* from *Enterobacteriaceae* isolates [51]. In all cases, detection occurred within 20–50 min and generally sensitivity was in the picogram range. In contrast, RPA offers a simplified system with fewer primers that generally gives results in less than 10 min, which may be a critical time advantage in certain settings like clinical applications. In contrast to LAMP, genomic detection of antimicrobial resistance by RPA is still in its infancy and more progress has been made toward identifying single nucleotide polymorphisms that convey drug resistance. In one study, an HIV drug resistance allele was detected by RPA combined with an oligonucleotide ligation assay [20]. Another study identified multidrug resistant tuberculosis sequence variants using a nested RPA approach [28].

A recent study demonstrated a Thin Film Transistor sensor for RPA that significantly accelerates readout time, using pH changes during DNA amplification as an electrical signal [52]. The molecular targets in that study are beta lactamases conferring resistance to cephalosporins and carbapenems, and detection was achieved within 2–5 min; however those data do not include tests for specificity of the assay nor measurement of antimicrobial resistance levels in the bacteria [52]. Nevertheless these results broadly support our finding that RPA is a superior approach to genomic antimicrobial resistance testing. Innovative readout technologies hold promise to further improve temporal performance of these assays beyond the 7–10 min detection times we demonstrate, while also providing more portable systems for point-of-care or field uses.

Our work is timely, given recent focus on the reservoirs of antimicrobial resistance genes (‘resistomes’) within oral [38, 53] and gut [54–56] microbial communities. Our RPA assay for *mef(A)* is highly sensitive (down to picogram levels), and this sensitivity may offer new diagnostic potential. However, the existence of antimicrobial resistance genes within commensal strains of the oral cavity even of healthy individuals [38, 53] raises concerns that a highly sensitive antibiotic-resistance test like ours may detect the genes when no infection is present. However, understanding the dynamics and inter-individual variation even in a healthy resistome is an important part of personalized medicine, which includes the microbiome [57–60] and associated mediators of antimicrobial resistance [61]. Because the microbiome is a dynamic entity in which antimicrobial resistance genes are shared among members [53], it is clinically vital to monitor levels of antibiotic resistance genes in commensal bacteria of healthy individuals that may contribute to more severe disease. For example, infections caused by cystic fibrosis are increasingly antibiotic resistant due to the horizontal transfer of resistance genes from commensal bacteria [62].

To date there is no cheap, easy, rapid assay to measure *mef(A)* in a patient’s healthy microbiome, but we provide such a tool, validated to show the genetic signature correlates with actual erythromycin resistance. Furthermore, having insight into the presence of resistance genes in the (healthy) microbiome of a patient would properly inform clinicians should that person become sick, reducing both morbidity and therapeutic failure and re-treatment. In other words, a patient with intrinsically high levels of *mef(A)* in her healthy microbiome would be best advised to avoid macrolide treatments if she becomes ill.

The question of whether our RPA assay would distinguish infection from colonization is related to a larger debate in the diagnostic field: when is a molecular assay too sensitive? Molecular detection methods like qPCR or RPA are much more sensitive than culture methods, often identifying many more microbes than culture [40, 63], leading some to conclude that the diagnostic utility of these methods is limited due to false positives [64]. However, there are several strategies for mitigating this risk: for example, testing only at-risk populations, as applied to testing for *C. difficile* or Group-A *Streptococcus* (*S. pyogenes)* [64]. This strategy minimizes the chance of a false-positive detection by not employing the test in cases unlikely to represent true infection. Thus, a clinician might deploy our new *mef(A)* assay when a patient exhibits symptoms consistent with bacterial infection, to guide choice of therapeutic agent. A second, and more powerful strategy is to focus on *levels* of the genetic sequence observed. If *mef(A)* is helping a pathogen cause disease, it will be enriched to a higher copy number than it would be as a sporadic colonizer diluted into a healthy microbial community [65, 66]. By providing quantitative data on relative levels of *mef(A),* our RPA assay is ideally suited to this approach, making the determination of an infection a matter of comparing the detected gene level with a threshold (after normalizing to total bacterial load). Critically, future work must focus on empirically setting the threshold by testing many clinical samples, from both healthy and sick patients [65]. By providing a validated, easy-to-use rapid molecular assay, the present study represents a vital first step in this process.

*Mef(A)* has been found in a wide variety of bacterial hosts [37], from *Neisseria gonorrhoeae* [67] to *Enterococcus faecalis* [68] and *Streptococcus pneumoniae* and *pyogenes* [33], and it has recently been found within commensal strains including *Streptoccous salivarius* [38] as we independently confirmed using RPA. We anticipate the *mef(A)* assay we validated in this work will become an important tool in the diagnostic toolbox, offering physicians and scientists alike a rapid, accurate measure of macrolide resistance, whether hosted in the upper (*S. pyogenes* [33] or *S. salivarius* [38]) or lower respiratory tract (*Streptococcus pneumoniae* [33] or *Staphylococcus aureus* [69] or others), or in other regions of the human microbiome.

## Abbreviations

LAMP: Loop-mediated isothermal AMPlification
mef(A): Macrolide Efflux protein A
MIC: Minimum Inhibitory Concentration
qPCR: Quantitative Polymerase Chain Reaction
RPA: Recombinase Polymerase Assay
